# Clinical and epidemiological characteristics of influenza and SARS-CoV-2 virus among patients with acute febrile illness in selected sites of Ethiopia 2021–2022

**DOI:** 10.3389/fpubh.2025.1549159

**Published:** 2025-07-09

**Authors:** Musse Tadesse Chekol, David Sugerman, Adamu Tayachew, Zelalem Mekuria, Neamin Tesfay, Aynalem Alemu, Andargachew Gashu, Wolde Shura, Melaku Gonta, Admikew Agune, Aster Hailemariam, Yonas Assefa, Mesfin Wossen, Abdulhafiz Hassen, Parsons Michele, Rachel Silver, Hulemenaw Delelegn, Lozano Briana, Tesfu Kasa, Nigatu Kebede

**Affiliations:** ^1^Public Health Emergency Management Center, Ethiopian Public Health Institute, Addis Ababa, Ethiopia; ^2^Aklilu Lemma Institute of Pathobiology, Addis Ababa University, Addis Ababa, Ethiopia; ^3^U.S. Centers for Disease Control and Prevention (CDC), Atlanta, GA, United States; ^4^Global One Health Initiative (GOHi), Ohio State University, Columbus, OH, United States

**Keywords:** AFI, influenza virus, SARS-CoV-2, Ethiopia, proportion

## Abstract

**Background:**

Viral respiratory pathogens have become the leading cause of acute undifferentiated febrile illness (AFI). We determined the fraction of AFI attributable to influenza and SARS-CoV-2 in Ethiopia, along with an understanding of their epidemiological characteristics.

**Methods:**

From February 2021 to June 2022, we enrolled patients meeting an AFI case definition (age >5 years with fever ≥38°C) who presented at one of four selected sentinel hospital sites in Jimma, Harari, Addis Ababa, and Gonder. Clinical and epidemiological information was collected, Nasopharyngeal swab samples were collected and analyzed using real-time PCR for respiratory viruses (influenza and SARS-CoV-2). A quasi-binomial regression model and multivariable regression were performed to compute fractions and establish associations with the agent detected.

**Result:**

A total of 737 AFI cases were enrolled. The overall proportion of SARS-CoV-2, influenza A, and influenza B among AFI patients were 7.8, 1.9, and 0.5 per 100,000 population, respectively. Among the enrolled AFI cases tested for SARS-CoV-2 and Influenza virus, SARS-CoV-2 was the most detected pathogen with a positivity rate of 13.7% (95% CI:11.3–16.4), followed by influenza A and influenza B, which have a positivity rate of 3.3% (95% CI: 2.2–5.1) and 0.8% (95% CI:0.3–1.8), respectively. The positivity rate of SARS-CoV-2 peaked at 37.4% in September 2021. Per the multivariable analysis, cases ≥65 years of age were three [AOR = 3.3,95% CI:(1.9–5.7)] times more likely to be positive for SARS-CoV-2.

**Conclusion:**

SARS-CoV-2 and influenza viruses were highly prevalent among AFI cases. The proportion of SARS-CoV-2 was higher among older adults. Further study is recommended to characterize influenza subtypes, SARS-CoV-2 variants and determine their attributable fraction among a broader panel of AFI-causing pathogens that contributes for guiding the proper diagnostics, treatment and surveillance measures.

## Introduction

Acute febrile illness (AFI) is one of the major reasons for outpatient visits and hospital admission among both children and adults (1, 2). AFI is typically characterized by fever without localizing manifestations, making it very challenging to confirm a diagnosis solely based on clinical history and physical examination (3). Beyond malaria, laboratory confirmation of other potential causes of AFI is expensive and complex, with unsatisfactory sensitivity and specificity (4, 5). To make things worse, resource-constrained settings have inadequate laboratory capacity to confirm suspected AFI cases hence practitioners utilize empirical management of cases (6). This approach has a far-reaching impact in exacerbating the risk of anti-microbial resistance (7).

The presence of considerable gaps in case definition, comparability of diagnostic assays, and control group to calculate attributable fractions were the major identified bottlenecks that hamper having a comparable estimate of the burden of AFI globally (8–10). Despite a significant reduction in the last two decades, malaria remains a common primary diagnosis in both malaria-endemic and non-endemic regions due to limited laboratory capacity to detect other AFI causing agents, and a lack of recognition of emerging and re-emerging etiologies as a potential differential diagnosis in consideration of local context (11–13). Among those emerging and re-emerging etiologies, viral respiratory tract infections are becoming the top cause of AFI (14).

Among viral respiratory infections, influenza A and B viruses are the most burdensome human respiratory pathogens (15). Depending on the circulating strain, annual seasonal influenza epidemics result in 290,000–650,000 deaths worldwide and affect up to 20% of the population (16). In 2020, a novel and virulent form of coronavirus emerged and was officially named by the World Health Organization (WHO) as SARS-CoV-2 (17). Due to the unprecedented speed of expansion that overwhelmed local capacity, WHO declared COVID-19 a global pandemic (18). As of 8^th^ September 2024, over 776. 2 million confirmed cases and over 7 million deaths have been reported globally (19). Worldwide, the presence of a notable gap in case-counting makes it difficult to make a comparison across countries (20). The surveillance systems in most African countries have gaps in diagnostic capacity, shortage of staffing, and poor data handling (21, 22). Ethiopia established an influenza sentinel surveillance system in 2008 to address these gaps and optimize pandemic preparedness efforts (23).

In Ethiopia, the positivity rate of influenza among suspected Influenza-like Illness (ILI) cases is estimated at up to 20% with a predominance of Influenza A subtype along with marked seasonal variation (24, 25). On the other hand, the national positivity rate of SARS-CoV-2 ranges from 6 to 9%, with significant variation by age, sex, residence, medical condition, and background prevalence (26–29). Nevertheless, the presence of the COVID-19 pandemic in Africa severely compromised the existing surveillance system (30). The Ethiopian routine disease surveillance system was not capable of capturing COVID-19 cases due to gaps in the community and event-based surveillance (31). This study aims to determine the fraction of those selected respiratory pathogens among suspected AFI cases and, in addition, the study’s objective was to understand their clinical and epidemiological characteristics during the time of the COVID-19 pandemic. This study is expected to provide a clearer picture on the proportion of respiratory pathogens as a cause of febrile illness in the study sites.

The finding of this study contributes for a more precise mapping of viral respiratory infections with acute fever, which will be used to develop evidence-based algorithms for the management of febrile illnesses associated with influenza and SARS-CoV-2, and to inform rational surveillance efforts in the future. It also provides information to policy makers and health care workers to consider proper diagnostic approaches and treatment measures (like influenza and SARS-CoV-2 vaccination) for the public at large in the future.

## Materials and methods

### Setting and study period

The study was conducted in four referral hospitals (sentinel sites) in Ethiopia: namely, Jimma University Hospital (JUH, Jimma), Gonder University Hospital (GUH, Gonder), Hiwot-Fana comprehensive specialized Hospital (HFH, Harar), and St. Paul’s Millenium Medical College Hospital (SPH, Addis Ababa). The selected health facilities are estimated to serve more than 8 million people. The sites were selected using an assessment checklist with predefined criteria, including geographic location, patient volume, the capacity of the laboratory to properly collect, handle, store, and transport specimens to the testing laboratory at Ethiopian Public Health Institute, availability of sentinel surveillance system, and willingness of the hospitals to collaborate in the implementation of project activities. The study was conducted from February 2021 to June 2022.

### AFI case enrollment

Inpatients and outpatients of any sex aged 5 years and above presenting at the selected facilities, and who met the case definition criteria for AFI, were eligible for enrollment in this study. The inclusion and exclusion criteria for the enrolled AFI cases were applied where the Inclusion criteria were patients aged ≥5 years old, measured axillary temperature ≥ 380c and experienced acute fever for 2–14 days. The exclusion criteria were subjects who do not consent to participate, subjects with localizing symptoms or identifiable focus of infection, subjects with chef complaint are injury or trauma; and obstetric related cases and surgical related underlying problems.

The sample size was calculated using a threshold approach (*n* = Z^2^*P*(1-P)/D^2^), with 95% CI, 0.05 precision value, and 20% expected prevalence (25), and the initial sample size was 480. To ensure representativeness and account for the effect of patient flow through the facilities, the sample size was doubled for each site, and cases were selected using a systematic sampling scheme where every nth case of AFI was enrolled. The nth value for each site was determined as the number of AFI cases seen by the facility divided by the maximum number of specimens that can be processed by the laboratory in a week. Samples from suspected AFI cases with inadequate specimens and incomplete data were excluded.

### Clinical and demographic data collection and testing procedures

Trained data collectors were assigned to each sentinel site. They collected demographic, clinical (e.g., symptoms, signs, treatment before enrollment), and epidemiological (e.g., exposures, travel history, significant medical/social history) information from eligible and enrolled cases by interviewing the patients and guardians. Clinical, epidemiologic, and laboratory data were entered electronically onto tablets using an Open Data Kit (ODK) platform. Both electronic and paper-based data collection mechanisms were used at each study site to make one method a backup for the other.

Nasopharyngeal swabs were collected from enrolled cases using a sterile COPAN brand universal transport medium containing 1–3 mL Viral Transport Media. The collected sample was vortexed and aliquoted in two cryovials for molecular testing at the National Influenza Center (NIC), Ethiopian Public Health Institute (EPHI). A specimen requisition form was filled out with basic demographic information, and the collected specimens were triple packaged and sent weekly with completed forms. Study sites were equipped with freezers or refrigerators throughout the study to temporarily store samples at 4°C until transported by trained postal service officers to the NIC at EPHI. EPHI’s NIC (laboratory) identified respiratory viruses from nasopharyngeal swabs. Nucleic acid was extracted from the swabs using the MagaBio plus Virus RNA Purification Kit II by MGISP-NE32 automated extractor. Real-time PCR was conducted on an ABI 7500FAST system (Life Technologies, Carlsbad, CA USA) using primers provided by CDC International Reagent Resource (CDC-IRR), a biological reagent repository established to provide better access to laboratory reagents.

### Statistical analysis

Statistical analyses were performed using R Studio version 4.2.2 and Stata version 17. Descriptive statistics were presented as medians, ranges, and interquartile ranges (IQR) for continuous variables and as proportions and charts for categorical variables. The proportion and positivity rate of respiratory infections associated with SARS-CoV-2 and influenza were determined to understand the epidemiology of the selected pathogens (73, 74). The proportion was computed by taking confirmed respiratory infection as a numerator, and the total outpatient visits and inpatient admissions of AFI cases as a denominator, whereas, in the case of positivity rate (PR) calculation, the denominator was changed to total AFI cases enrolled in the study while the numerator remained unchanged. The proportion was presented per 100,000 cases, while the PR was presented by percentage.

A quasi-binomial regression model was used to compute the fraction of SARS-CoV-2 and Influenza that was associated with AFI. Finally, the association between clinical and epidemiologic characteristics and testing positive for selected pathogens were further evaluated using a multivariable logistic regression approach.

### Ethical clearance

Ethical clearance for this study was obtained from EPHI’s Scientific and Ethical Review Office (SERO) and Addis Ababa University (EPHI-IRB-254-2020; MM No. 065). Written informed consent was obtained from each participant or guardian (for pediatric patients aged 5–12 years old) prior to the enrollment in the study for an interview and sample collection. Participants were identified by coded study numbers in all data collection forms and electronic databases. No individual identifiers were used in any reports.

## Result

### Patient screening and enrollment

During the study period, a total of 737 eligible cases were enrolled, among them, 29.3, 26.8, 24.6, and 19.1% were enrolled from Addis Ababa, Gonder, Jimma, and Harari, respectively. Most enrolled participants were male (61.7%) and inpatient cases (66.3%). The median age of enrolled cases was 37 years, with a minimum age of 5 years to a maximum of 95 years (Table 1). Furthermore, only 19.0% of cases took fever-reducing medication before seeking care, and 48% of the cases had had a recorded temperature >38°C at the time of initial diagnosis.

**Table 1:** Demographic and clinical characteristics of enrolled cases included in the study by site in Ethiopia.

Characteristic	Overall (*N* = 737) (%)	GUH (*n* = 198) (%)	HFH (*n* = 141) (%)	JUH (*n* = 182) (%)	SPH (*n* = 216) (%)
Age group					
Median (IQR)	37(25–55)	29(22–42)	28(22–36)	47(30–60)	45(30–60)
5–14	59(8.0)	16(8.1)	10(7.1)	7(3.9)	26(12.0)
15–24	118(16.0)	42(21.2)	38(27.0)	26(14.3)	12(5.6)
25–44	272(36.9)	98(49.5)	66(46.8)	42(23.1)	66(39.6)
45–64	192(26.1)	32(16.2)	25(17.3)	70(38.5)	65(30.1)
65+	96(13.0)	10(5.1)	2(1.4)	37(20.3)	47(21.8)
Sex					
Female	282(38.3)	83(41.9)	45(31.9)	59(32.4)	95(44.0)
Male	455(61.7)	115(58.1)	96(68.1)	123(67.6)	121(56.)
Case type					
Outpatient	489(66.3)	112(56.6)	118(83.7)	146(80.2)	113(52.3)
Inpatient	248(33.7)	86(43.3)	23(16.3)	36(19.8)	103(47.7)
Taking fever-reducing medication					
Yes	140(19.0)	74(37.4)	40(28.4)	0(0.0)	26(12.1)
No	597(81.0)	124(62.6)	101(71.6)	182(100.0)	190(87.9)

**Figure 1:**
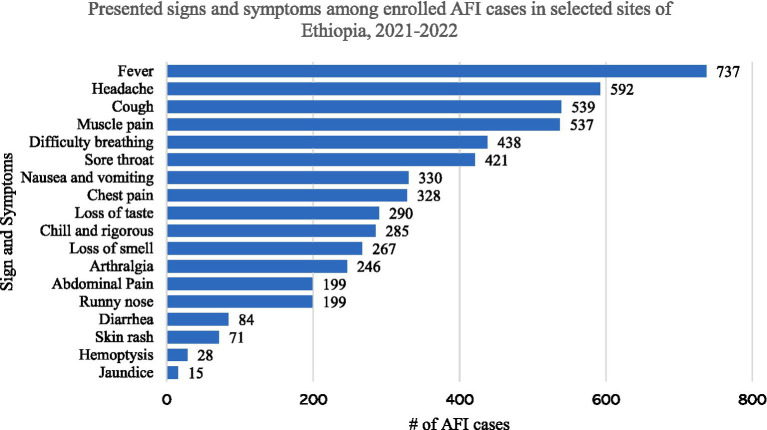
Presented signs and symptoms among enrolled cases in Ethiopia.

The most common presenting complaints, other than fever, were headache (80.3), cough (73.1%), muscle and joint pain (72.9%), and difficulty of breathing (59.4%). The least common presenting complaints were diarrhea (11.4%), skin rash (9.6%), hemoptysis (3.8%) and jaundice (2.0%) (Figure 1).

When examined by study site, 92.4 and 88.9% of cases from GUH presented with headache and muscle pain, while 84.1% of enrolled cases from JUH presented with cough (Table 2). Furthermore, 61.2, 17.6, and 7.6% of enrolled cases were primarily diagnosed as respiratory tract infections, malaria, and gastrointestinal tract infections, respectively. Geographically, 79.2% of cases from SPH and 73.6% of the cases from JUH were primarily diagnosed as respiratory tract infections, whereas 53.5% of cases from GUH were diagnosed as malaria. With respect to possible risk factors, 21.9, 5.3, and 5.2% of cases had a comorbid illness, contact with a dead animal, and travel history, respectively (Table 2).

**Table 2:** Clinical manifestations, tentative diagnosis, and possible risk factor of enrolled cases by the site in Ethiopia.

Characteristic	Overall (*N* = 737) (%)	GUH (*n* = 198) (%)	HFH (*n* = 141) (%)	JUH (*n* = 182) (%)	SPH (*n* = 216) (%)
Sign and symptoms					
Cough	539(73.1)	145(73.2)	107(75.9)	153(84.1)	134(73.1)
Sore throat	421(57.2)	125(63.1)	75(53.2)	99(54.4)	122(56.5)
Difficulty of breathing	438(59.4)	103(52.1)	84(59.6)	110(60.4)	141(65.3)
Hemoptysis	28(3.8)	23(11.6)	3(2.1)	1(0.6)	1(0.5)
Runny nose	199(27.0)	47(23.7)	48(34.0)	64(35.2)	40(18.5)
Diarrhea	84(11.4)	46(23.2)	24(17.1)	2(1.1)	12(5.6)
Nausea and vomiting	330(44.8)	145(73.2)	63(44.7)	30(16.5)	92(42.6)
Abdominal Pain	199(27.0)	82 (41.4)	69 (48.9)	8 (4.4)	40(18.5)
Skin rash	71(9.6)	13(17.7)	13(9.2)	5(2.8)	18(8.3)
Headache	592(80.3)	183(92.4)	115(81.6)	158(86.8)	136(62.9)
Arthralgia	246(33.4)	153(77.3)	8(5.7)	30(16.5)	55(25.5)
Muscle and joint pain	537(72.9)	176(88.9)	101(71.6)	127(69.8)	133(61.6)
Chest pain	328(44.5)	87(43.9)	77(54.6)	128(70.3)	36(16.7)
Loss of taste	290(39.4)	90(45.5)	77(54.6)	117(64.3)	6(2.8)
Loss of smell	267(36.2)	85(42.9)	65(46.1)	111(61.0)	6(2.8)
Chill and rigors	285(38.7)	98(49.5)	58(41.1)	37(20.3)	92(42.6)
Jaundice	15(2.0)	11(5.6)	3(2.1)	0(0.0)	1(0.5)
Primary clinical diagnosis					
Respiratory tract infection	451(61.2)	77(38.9)	69(48.9)	134(73.6)	171(79.2)
Malaria	130(17.6)	106(53.5)	5(3.6)	14(7.7)	5(2.3)
Gastrointestinal tract infection	56(7.6)	39(1.5)	31(22.0)	18(9.9)	4(1.9)
Central nervous system infection	13(1.8)	2(1.0)	7(5.0)	4(2.2)	0(0.0)
Cardiovascular disease	12(1.6)	1(0.5)	5(3.6)	5(2.8)	1(0.5)
Other choric medical illness	9(1.2)	3(1.5)	1(0.7)	1(0.6)	4(1.9)
Acute abdomen	7(1.0)	0(0.0)	4(2.80)	0(0.0)	3(1.4)
Metabolic disorder	7(1.0)	0(0.0)	1(0.7)	1(0.6)	5(2.3)
Urinary tract infection	4(0.5)	0(0.0)	0(0.0)	0(0.0)	4(1.9)
HIV	4(0.5)	0(0.0)	0(0.0)	2(1.1)	2(0.9)
Chronic bronchitis	3(0.4)	0(0.0)	1(0.7)	2(1.1)	0(0.0)
Poisoning	2(0.3)	0(0.0)	1(0.7)	0(0.0)	1(0.5)
Bleeding disorder	2(0.3)	0(0.0)	1(0.7)	0(0.0)	1(0.5)
Others	37(5.0)	6(3.0)	15(10.6)	1(0.6)	15(6.9)
Risk factors					
Having comorbid illness	161(21.9)	12(6.1)	26(18.4)	79(43.4)	44(20.4)
Close contact suspected cases	26(3.5)	6(3.0)	5(3.6)	2(1.1)	13(6.0)
Close contact with dead animals	39(5.3)	16(8.1)	2(1.4)	15(8.2)	6(2.8)
History of travel	38(5.2)	20(10.1)	14(9.9)	1(0.6)	3(1.4)
Admitted to ICU	21(2.9)	8(4.0)	1(0.7)	9(5.0)	3(1.4)

### Epidemiological description of confirmed cases

**Figure 2:**
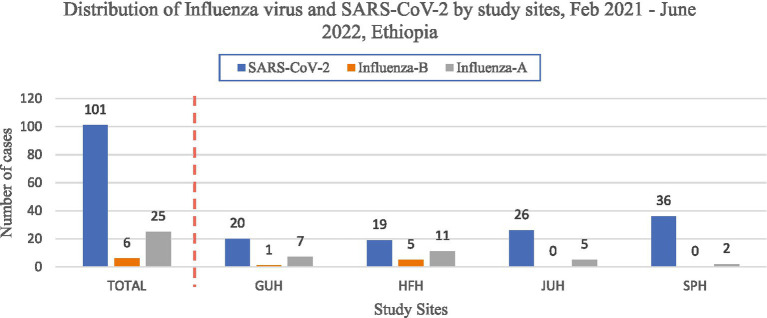
Distribution of influenza and SARS-COV-2 cases by study site in Ethiopia.

A total of 132(17.9%) cases were positive for Influenza and SARS-CoV-2 respiratory viruses (Figure 2).

The most prevalent respiratory pathogen was SARS-CoV-2 with a positivity rate of 13.7% followed by influenza A and influenza B, which had positivity rates of 3.3 and 0.8%, respectively. The positivity rate of SARS-CoV-2 was higher among cases from St. Paul Hospital (SPH) in Addis Ababa (16.7%), males (15.2%), and age >65 years (30.2%). Furthermore, influenza A virus was higher among cases from Hiwot-Fana Hospital (HFH) in Harar (7.8%), females (5.3%), and age 15–24 years (6.8%). With respect to proportion of influenza and SARS-CoV-2, the rate was higher among those ≥ 65 years (17.1 per 100,000 population), males (9.8 per 100,000 population), and Harar site (13.3 per 100,000 population) as compared to their respective categories (Table 3).

**Table 3:** Proportion and positivity rate of influenzas and SARS_CoV-2 by age, sex, and site in Ethiopia.

Variable	Category	Total cases visited the sites	Total enrolled case	Influenza A Virus (95%CI)	Influenza B Virus (95%CI)	SARS-CoV-2 Virus (95%CI)	PR of Influenza A Virus (95%CI)	PR of Influenza B Virus (95%CI)	PR of SARS-CoV-2 Virus (95%CI)
Age group	5–14y	91,657	59	1.1 (0.1–7.1)	1.1 (0.1–7.1)	1.1 (0.1–7.1)	1.7 (0.9–10.2)	1.7 (0.9–10.2)	1.7 (0.9–10.2)
	15–24y	403,251	118	2.0 (0.9–4.1)	0.3 (0.01–1.6)	2.9 (1.6–5.4)	6.8 (3.2–13.3)	0.8 (0.04–5.3)	10.2 (5.6–17.4)
	25–44y	319,462	272	2.8 (1.4–5.6)	_	9.7 (6.7–13.9)	3.3 (1.6–6.4)	_	11.3 (7.9–15.9)
	45–65y	295,642	192	1.1 (0.3–3.2)	1.3 (0.4–3.7)	9.5 (6.4–13.8)	1.5 (0.4–4.8)	2.1 (0.6–5.5)	14.5 (10.1–20.5)
	>65y	170,874	96	2.3 (0.7–6.4)		17.(11.524)	4.1(1.3–11.0)	_	30.2 (21.4–40.5)
Sex	Male	586,253	455	1.7 (0.9–3.3)	1.1 (0.4–2.3)	9.8 (7.6–12.8)	2.2 (1.1–4.1)	1.3 (0.5–2.9)	15.2 (11.3–20.1)
	Female	694,633	282	2.2 (1.3–3.7)	_	6.2 (4.5–8.4)	5.3 (3.1–8.8)	___	12.7 (9.8–16.2)
Study Site	SPH	483,216	216	0.4 (0.07–1.7)	_	7.4 (5.3–10.4)	0.9 (0.1–3.6)	_	16.7 (12.1–22.2)
	JUH	296,751	182	1.7 (0.6–4.8)	_	8.7 (5.8–13.0)	2.7 (1.1–6.6)	1.5 (1.3–8.5)	14.3 (9.7–20.4)
	HFH	142,756	141	7.7 (4.1–14.2)	3.5 (1.3–8.7)	13.3 (8.2–21.2)	7.8 (4.1–13.8)	_	13.9 (8.5–20.5)
	GUH	358,163	198	2.0(0.9–4.2)	0.3(0.02–1.8)	5.5 (3.5–8.7)	6.7 (3.1–13.3)	0.5 (0.02–3.2)	10.1(6.4–15.3)
Total		1,280,886	737	1.9 (1.3–2.9)	0.5 (0.2–1.1)	7.8 (6.5–9.6)	3.3 (2.2–5.1)	0.8 (0.3–1.8)	13.7 (11.3–16.4)

**Figure 3:**
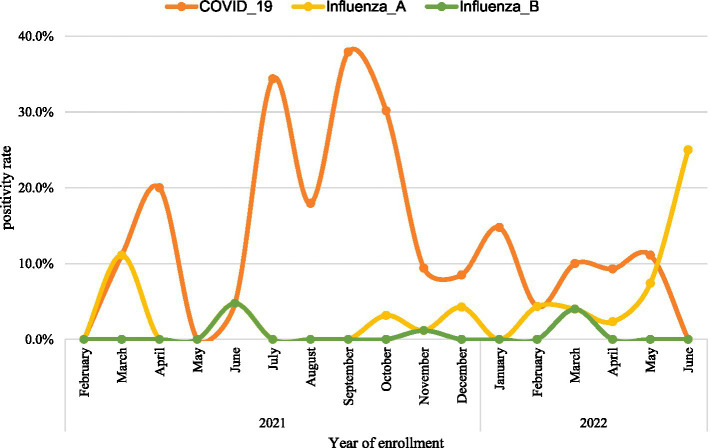
The trend of the positivity rate of influenza and SARS_COV-2 from 2021 to 2022 in Ethiopia.

The positivity rates of SARS_CoV-2 markedly varied across months of the year, the positivity rate peaked during 2021 in September (37.9%), July (34.4%), and April (20.0%). The positivity rate of influenza A sharply increased in 2022, from 2.3% in April to 25% in June (Figure 3).

When we looked at age group and clinical manifestation, cases aged 65 and above, were 3 times [AOR = 3.3,95%CI (1.9–5.7)] more likely to be positive for SARS-CoV-2 as compared to cases aged between 25 to 44. Moreover, chest pain [AOR = 0.6,95%CI (0.3–0.9)] and chill and rigorous [AOR = 0.5,95%CI (0.3–0.8)] were negatively associated with SARS-CoV-2 (Table 4).

**Table 4:** Association of age group and clinical features of overall study participants with SARS_CoV-2.

Risk factors	Adjusted odds ratio	95% Confidence Interval	*P-*value
Age group			
5–14	0.3	0.1–1.0	0.06
15–24	1.1	0.6–2.0	0.78
25–44 ^r^	1		
45–64	1.4	0.8–2.2	0.25
65+	3.3	1.9–5.7	0.001
Symptoms			
Chest pain	0.6	0.3–0.9	0.040
Chill and rigorous	0.5	0.3–0.8	0.002

^r^ Reference group.

## Discussion

This study provides a deeper insight into the epidemiological and clinical manifestation of selected respiratory pathogens among AFI patients across four hospitals in Ethiopia. A standardized case definition of AFI were used for the enrolled cases with an inclusion and exclusion criteria and the case definition were not specific for respiratory cases. The proportion and positivity rate of SARS-CoV-2 was four-fold higher than influenza A and nearly 10 times higher than influenza B. Furthermore, marked variation in the positivity rate was observed in terms of sex, age group, site, and season.

The overall positivity rate of SARS-CoV-2 was 13.7%. The finding was comparable with studies conducted in India (32), and Pakistan (33); however, the positivity rate was much lower as compared to studies conducted in Tanzania (34), Kenya (35), Uganda (36) and Madagascar (37). The variation in the SARS-CoV-2 positivity rate may be explained by the method of laboratory investigation, study population, changes in the COVID-19 pandemic due to the introduction of new variants and vaccination as well as the level of preventive measures put forward in a country. Overall, tracking positivity rate is critical in monitoring the implemented mitigation measures along with paving the way for taking additional interventions to contain the pandemic (36).

This study elucidated that the proportion and positivity rate of SARS-CoV-2 was slightly higher among males as compared to females. The finding was parallel with studies conducted in other countries (38–41). This could be explained since males have lower health seeking behavior, so only present for care when sicker and more likely to test positive. Furthermore, their occupation and social engagements makes them more vulnerable to having a higher viral load resulting in disease severity, and mortality (42). Males are also thought to adhere to prescribed preventive measures less frequently than females (43). Thus, targeted communication to higher risk groups should be adopted and put in place for the proper implementation of the designed interventions.

The study revealed that the positivity rate of SARS-CoV-2 was much higher among old-aged cases (age ≥65 years) when compared with other age groups. This finding is corroborated by studies conducted elsewhere (44–47). Further exploring the risk of age with SARS-CoV-2 infection might be necessary to identify the exact correlation. Perhaps, older people are usually attached to low socioeconomic status, which has a role in contracting the illness due to a lack of hygienic measures like frequent hand washing and keeping physical distance (48). However, the preventive measures put in place to control the pandemic have resulted in untoward consequence on the health of the people (i.e., physical, mental, emotional, and social) and the economy of the world (49). Overall, control measures should be in place by evaluating the untoward consequences of those measures among the older population vis-à-vis outweighing the severity of the illness.

The positivity rate of SARS-CoV-2 reached its peak between July and September 2021. This finding concurred with other studies conducted (50, 51). Different geographic arrangements could account for different changes in the pandemic’s dynamics. For example, in African countries, the rapid rise in the positivity rate is linked to the emergence of new variants, such as the Beta and Delta variants (52). In summary, the findings imply a need for regular monitoring of the positivity rate of SARS-CoV-2 and influenza virus through the routine surveillance system where the surveillance data and specimens collected on a regular manner and reported via the Integrated Disease Surveillance System (IDSR) accompanied by *ad hoc* surveillance to take timely measures.

In this study, chills and rigor as well as chest pain were not indicative symptoms for the diagnosis of SARS-CoV-2. This finding agreed with studies conducted elsewhere (53–55). Chest pain is commonly attached to major adverse cardiac events such as coronary artery disease (56–58), while chills and rigor are usually indicative diagnosis of arboviral and other acute respiratory tract infections (59–62). Thus, health professionals should be vigilant enough to consider other diagnoses, other than SARS-CoV-2, when they encounter chills, rigor, and chest pain while taking a clinical history of cases.

In this study, both PR and proportion of SARS-CoV-2 were higher in HFH and SPH as compared to other sites. A similar finding was also reported from studies conducted in Spain and Kenya, which indicated that the burden of SARS-CoV-2 was much higher in major cities (63, 64). Major cities have unique characteristics explained by high population density and road connectivity, which are fertile ground for rapid transmission of the pandemic due to the increased chance of physical contact (65, 66). For effective containment of SARS-CoV-2, measures should be taken to raise public awareness of physical distancing in public places.

With respect to the overall proportion and positivity rate of influenza virus, influenza A was predominant over influenza B. However, in Ethiopia, the positivity rate observed during this study period was five folds lower than when compared before the pandemic, which reached up to 20% (24, 25). This could be explained by alterations in the epidemiology of the virus during the course of the pandemic (67). Non-pharmaceutical Interventions (NPI) implemented to curb the burden of the pandemic have resulted in a reduction in hospitalization and mortality due to Non-SARS-CoV-2 (68–70). Although the burden of influenza notably reduced, the reduction during the pandemic has raised a tone of questions that need to be addressed related to seasonality and preventative measures (71). On top of this, the pandemic has impacted the health system (72). The combination of all these could result in the rebound of influenza and could left the world in a precarious public health position that warrants weighing potential pandemic risks more seriously.

This study and its results are subject to the following limitations. RT-PCR misses the detection of people with SARS-CoV-2 infection unless cases present during the acute phase of illness when viremia is high and detectable by RT-PCR assay. This hinders to enroll the actual sample size and could introduce a substantial risk of bias by underestimating the positivity rate. In addition, case enrollment across all months/season were not consistent. As a result, seasonal variations in the occurrence of influenza and SARS-CoV-2; and other AFI causing pathogens were not ruled out by laboratory testing in this study.

## Conclusion

SARS-CoV-2 and influenza viruses were highly prevalent in AFI cases in Ethiopia. The proportion of SARS-CoV-2 was highest among persons aged over 65 years of age. A further study is recommended to explore influenza subtypes and SARS-CoV-2 variants among cases and determine their influence on disease severity, signs and symptoms, and on the fraction of broader pathogens causing AFI in Ethiopia so as to provide evidence-based information that guides a proper diagnosis, clinical care and surveillance approaches.

## Data Availability

The raw data supporting the conclusions of this article will be made available by the authors, without undue reservation.

## References

[1] Prasad N Sharples KJ Murdoch DR Crump JA. Community prevalence of fever and relationship with malaria among infants and children in low-resource areas. Am J Tropical Med Hygiene. (2015) 93:178–80. doi: 10.4269/ajtmh.14-0646, PMID: 25918207 PMC4497891

[2] Crump JA Kirk MD. Estimating the burden of febrile illnesses. PLoS Negl Trop Dis. (2015) 9:e0004040. doi: 10.1371/journal.pntd.0004040, PMID: 26633014 PMC4668833

[3] Snow RW Eckert E Teklehaimanot A. Estimating the needs for artesunate-based combination therapy for malaria case management in Africa. Trends Parasitol. (2003) 19:363–9. doi: 10.1016/s1471-4922(03)00168-5, PMID: 12901938

[4] Peeling RW Fongwen N. Solving the enigma of acute febrile illness. Lancet Infect Dis. (2022) 22:1261–2. doi: 10.1016/S1473-3099(22)00313-9, PMID: 35716699

[5] Crump JA Gove S Parry CM. Management of adolescents and adults with febrile illness in resource-limited areas. BMJ. (2011) 343:d4847. doi: 10.1136/bmj.d4847, PMID: 21824901 PMC3164889

[6] Hercik C Cosmas L Mogeni OD Wamola N Kohi W Omballa V. A diagnostic and epidemiologic investigation of acute febrile illness (AFI) in Kilombero, Tanzania. PLoS One. (2017) 12:e0189712. doi: 10.1371/journal.pone.0189712, PMID: 29287070 PMC5747442

[7] Murray CJ Ikuta KS Sharara F Swetschinski L Aguilar GR Gray A. Global burden of bacterial antimicrobial resistance in 2019: a systematic analysis. Lancet. (2022) 399:629–55. doi: 10.1016/S0140-6736(21)02724-0 35065702 PMC8841637

[8] Rhee C Kharod GA Schaad N Furukawa NW Vora NM Blaney DD. Global knowledge gaps in acute febrile illness etiologic investigations: a scoping review. PLoS Negl Trop Dis. (2019) 13:e0007792. doi: 10.1371/journal.pntd.0007792, PMID: 31730635 PMC6881070

[9] Hopkins H Bruxvoort KJ Cairns ME Chandler CI Leurent B Ansah EK. Impact of introduction of rapid diagnostic tests for malaria on antibiotic prescribing: analysis of observational and randomized studies in public and private healthcare settings. BMJ. (2017) 356:j1054. doi: 10.1136/bmj.j1054, PMID: 28356302 PMC5370398

[10] Bressan CD Teixeira MD Gouvêa MI de Pina-Costa A Santos HF Calvet GA. Challenges of acute febrile illness diagnosis in a national infectious diseases center in Rio de Janeiro: 16-year experience of syndromic surveillance. PLoS Negl Trop Dis. (2023) 17:e0011232. doi: 10.1371/journal.pntd.0011232, PMID: 37011087 PMC10101631

[11] Bhowmick IP Pandey A Subbarao SK Pebam R Majumder T Nath A. Diagnosis of indigenous non-malarial vector-borne infections from malaria negative samples from community and rural hospital surveillance in Dhalai District, Tripura, north-East India. Diagnostics. (2022) 12:362. doi: 10.3390/diagnostics12020362, PMID: 35204453 PMC8871021

[12] Iroh Tam PY Obaro SK Storch G. Challenges in the etiology and diagnosis of acute febrile illness in children in low-and middle-income countries. J Pediatr Infect Dis Soc. (2016) 5:190–205. doi: 10.1093/jpids/piw016, PMID: 27059657 PMC7107506

[13] Crump JA Morrissey AB Nicholson WL Massung RF Stoddard RA Galloway RL. Etiology of severe non-malaria febrile illness in northern Tanzania: a prospective cohort study. PLoS Negl Trop Dis. (2013) 7:e2324. doi: 10.1371/journal.pntd.0002324, PMID: 23875053 PMC3715424

[14] Kaboré B Post A Lompo P Bognini JD Diallo S Kam BT. Aetiology of acute febrile illness in children in a high malaria transmission area in West Africa. Clin Microbiol Infect. (2021) 27:590–6. doi: 10.1016/j.cmi.2020.05.029 32505586

[15] Sharma Y Horwood C Hakendorf P Thompson C. Clinical characteristics and outcomes of influenza A and B virus infection in adult Australian hospitalized patients. BMC Infect Dis. (2020) 20:1–9. doi: 10.1186/s12879-020-05670-8 PMC7705848 33261559

[16] Tyrrell CS Allen JL Gkrania-Klotsas E. Influenza: epidemiology and hospital management. Medicine. (2021) 49:797–804. doi: 10.1016/j.mpmed.2021.09.015, PMID: 34849086 PMC8624711

[17] Huang C Wang Y Li X Ren L Zhao J Hu Y. Clinical features of patients infected with 2019 novel coronavirus in Wuhan, China. Lancet. (2020) 395:497–506. doi: 10.1016/S0140-6736(20)30183-5, PMID: 31986264 PMC7159299

[18] Cucinotta D Vanelli M. WHO declares COVID-19 a pandemic. Acta Bio Medica Atenei Parmensis. (2020) 91:157. doi: 10.23750/abm.v91i1.9397, PMID: 32191675 PMC7569573

[19] World Health Organization. (2023). COVID-19 weekly epidemiological update, edition 171, 2024. World Health Organization. Available online at: https://www.who.int/emergencies/diseases/novel-coronavirus-2019/situation-reports (Accessed December 22, 2024).

[20] Alvarez E Bielska IA Hopkins S Belal AA Goldstein DM Slick J. Limitations of COVID-19 testing and case data for evidence-informed health policy and practice. Health Res Policy Syst. (2023) 21:11. doi: 10.1186/s12961-023-00963-1, PMID: 36698202 PMC9876649

[21] Adebisi YA Rabe A Lucero-Prisno DE III. COVID-19 surveillance systems in African countries. Health Promotion Perspectives. (2021) 11:382–92. doi: 10.34172/hpp.2021.49, PMID: 35079582 PMC8767077

[22] Fawole OI Bello S Adebowale AS Bamgboye EA Salawu MM Afolabi RF. COVID-19 surveillance in Democratic Republic of Congo, Nigeria, Senegal and Uganda: strengths, weaknesses, and key lessons. BMC Public Health. (2023) 23:1–5. doi: 10.1186/s12889-023-15708-6 37158897 PMC10165588

[23] Ayele W Demissie G Kassa W Zemelak E Afework A Amare B. Challenges of establishing routine influenza sentinel surveillance in Ethiopia, 2008–2010. J Infect Dis. (2012) 206:S41–5. doi: 10.1093/infdis/jis531 23169970

[24] Woyessa AB Mengesha M Belay D Tayachew A Ayele W Beyene B. Epidemiology of influenza in Ethiopia: findings from influenza sentinel surveillance and respiratory infection outbreak investigations, 2009–2015. BMC Infect Dis. (2018) 18:1–11. doi: 10.1186/s12879-018-3365-5 30176806 PMC6122732

[25] Tadesse M Mengesha M Tayachew A Belay D Hassen A Woyessa AB. Burden and seasonality of medically attended influenza like illness (ILI) in Ethiopia, 2012 to 2017. BMC Infect Dis. (2020) 20:1–3. doi: 10.1186/s12879-020-4827-0 PMC7029599 32070275

[26] Gudina EK Gobena D Debela T Yilma D Girma T Mekonnen Z. COVID-19 in Oromia region of Ethiopia: a review of the first 6 months’ surveillance data. BMJ Open. (2021) 11:e046764. doi: 10.1136/bmjopen-2020-046764, PMID: 33782023 PMC8008954

[27] Gedefie A Tilahun M Fiseha M Alemayehu E Shibabaw A Bisetegn H. Epidemiology of SARS-CoV-2 infection in Ethiopia: a systematic review and meta-analysis. COVID. (2023) 3:703–14. doi: 10.3390/covid3050052

[28] Muleta D Simieneh A Duguma T Tekalign E Worku T Ayele G. SARS-CoV-2 infections, clinical characteristics, and related risk factors: the first 8 months surveillance study conducted in Southwest Ethiopia. Inquiry. (2023) 60:00469580231166794. doi: 10.1177/00469580231166794, PMID: 37077149 PMC10119653

[29] Adane T Adugna Y Aynalem M. Prevalence of COVID-19 in West Gondar zone, Northwest Ethiopia: a population-based retrospective study. Disaster Med Public Health Prep. (2023) 17:e156. doi: 10.1017/dmp.2022.72, PMID: 35317876 PMC9095846

[30] Aborode AT Hasan MM Jain S Okereke M Adedeji OJ Karra-Aly A. Impact of poor disease surveillance system on COVID-19 response in Africa: time to rethink and rebuilt. Clin Epidemiol Global Health. (2021) 12:100841. doi: 10.1016/j.cegh.2021.100841, PMID: 34368503 PMC8330137

[31] Tamiru A Regassa B Alemu T Begna Z. The performance of COVID-19 surveillance system as timely containment strategy in Western Oromia, Ethiopia. BMC Public Health. (2021) 21:1–4. doi: 10.1186/s12889-021-12380-6 34922501 PMC8684163

[32] Inbaraj LR George CE Chandrasingh S. Seroprevalence of COVID-19 infection in a rural district of South India: a population-based sero epidemiological study. PLoS One. (2021) 16:e0249247. doi: 10.1371/journal.pone.0249247, PMID: 33788873 PMC8011723

[33] Ahmad AM Shahzad K Masood M Umar M Abbasi F Hafeez A. COVID-19 seroprevalence in Pakistan: a cross-sectional study. BMJ Open. (2022) 12:e055381. doi: 10.1136/bmjopen-2021-055381, PMID: 35387815 PMC8987211

[34] Nyawale HA Moremi N Mohamed M Njwalila J Silago V Krone M. High Seroprevalence of SARS-CoV-2 in Mwanza, northwestern Tanzania: a population-based survey. Int J Environ Res Public Health. (2022) 19:11664. doi: 10.3390/ijerph191811664, PMID: 36141938 PMC9517516

[35] Etyang AO Adetifa I Omore R Misore T Ziraba AK Ng’oda MA. SARS-CoV-2 seroprevalence in three Kenyan health and demographic surveillance sites, December 2020-May 2021. PLoS Glob Public Health. (2022) 2:e0000883. doi: 10.1371/journal.pgph.0000883 36962821 PMC10021917

[36] Al Dallal A AlDallal U Al Dallal J. Positivity rate: an indicator for the spread of COVID-19. Curr Med Res Opin. (2021) 37:2067–76. doi: 10.1080/03007995.2021.1980868, PMID: 34517740

[37] Schoenhals M Rabenindrina N Rakotondramanga JM Dussart P Randremanana R Heraud JM. SARS-CoV-2 antibody seroprevalence follow-up in Malagasy blood donors during the 2020 COVID-19 epidemic. EBioMedicine. (2021) 68:103419. doi: 10.1016/j.ebiom.2021.103419 34098337 PMC8176015

[38] Bassi F Arbia G Falorsi PD. Observed and estimated prevalence of COVID-19 in Italy: how to estimate the total cases from medical swabs data. Sci Total Environ. (2021) 764:142799. doi: 10.1016/j.scitotenv.2020.142799, PMID: 33066965 PMC7543749

[39] Javed W Abidi SH Baqar JB. Seroprevalence and characteristics of coronavirus disease (COVID-19) in workers with non-specific disease symptoms. BMC Infect Dis. (2022) 22:1–8. doi: 10.1186/s12879-022-07461-9 35596145 PMC9120800

[40] Abate BB Kassie AM Kassaw MW Aragie TG Masresha SA. Sex difference in coronavirus disease (COVID-19): a systematic review and meta-analysis. BMJ Open. (2020) 10:e040129. doi: 10.1136/bmjopen-2020-040129, PMID: 33028563 PMC7539579

[41] Tunheim G Rø GØ Tran T Kran AM Andersen JT Vaage EB. Trends in seroprevalence of SARS-CoV-2 and infection fatality rate in the Norwegian population through the first year of the COVID-19 pandemic. Influenza Other Respir Viruses. (2022) 16:204–12. doi: 10.1111/irv.12932 34751488 PMC8652705

[42] Pradhan A Olsson PE. Sex differences in severity and mortality from COVID-19: are males more vulnerable? Biol Sex Differ. (2020) 11:1. doi: 10.1186/s13293-020-00330-7 32948238 PMC7498997

[43] Bwire GM. Coronavirus: why men are more vulnerable to Covid-19 than women? SN Comprehens Clin Med. (2020) 2:874–6. doi: 10.1007/s42399-020-00341-w, PMID: 32838138 PMC7271824

[44] Herlinda O Bella A Kusnadi G Swasthika Nurshadrina D Thoriq Akbar M Nida S. Seroprevalence of antibodies against SARS-Cov-2 in the high impacted sub-district in Jakarta, Indonesia. PLoS One. (2021) 16:e0261931. doi: 10.1371/journal.pone.0261931, PMID: 34941968 PMC8699601

[45] Abul Y Leeder C Gravenstein S. Epidemiology and clinical presentation of COVID-19 in older adults. Infect Dis Clin N Am. (2023) 37:1–26. doi: 10.1016/j.idc.2022.11.001, PMID: 36805007 PMC9633621

[46] Setiadi W Rozi IE Safari D Daningrat WO Johar E Yohan B. Prevalence and epidemiological characteristics of COVID-19 after one year of pandemic in Jakarta and neighbouring areas, Indonesia: a single center study. PLoS One. (2022) 17:e0268241. doi: 10.1371/journal.pone.0268241, PMID: 35550635 PMC9098020

[47] Damayanthi HD Prabani KI Weerasekara I. Factors associated for mortality of older people with COVID-19: a systematic review and meta-analysis. Gerontol Geriatr Med. (2021) 7:23337214211057392. doi: 10.1177/23337214211057392, PMID: 34888405 PMC8649451

[48] Crimmins EM. Age-related vulnerability to coronavirus disease 2019 (COVID-19): biological, contextual, and policy-related factors. Public Policy Aging Report. (2020) 30:142–6. doi: 10.1093/ppar/praa023, PMID: 33214754 PMC7499698

[49] Cocuzzo B Wrench A O’Malley C. Effects of COVID-19 on older adults: physical, mental, emotional, social, and financial problems seen and unseen. Cureus. (2022) 14:3–7. doi: 10.7759/cureus.29493, PMID: 36299954 PMC9588279

[50] Bergeri I Whelan MG Ware H Subissi L Nardone A Lewis HC. Global SARS-CoV-2 seroprevalence from January 2020 to April 2022: a systematic review and meta-analysis of standardized population-based studies. PLoS Med. (2022) 19:e1004107. doi: 10.1371/journal.pmed.1004107, PMID: 36355774 PMC9648705

[51] Lyimo E Fougeroux C Malabeja A Mbwana J Hayuma PM Liheluka E. Seroprevalence of SARS-CoV-2 antibodies among children and adolescents recruited in a malariometric survey in North-Eastern Tanzania July 2021. BMC Infect Dis. (2022) 22:846. doi: 10.1186/s12879-022-07820-6, PMID: 36371172 PMC9652923

[52] Lewis HC Ware H Whelan M Subissi L Li Z Ma X. SARS-CoV-2 infection in Africa: a systematic review and meta-analysis of standardised seroprevalence studies, from January 2020 to December 2021. BMJ Glob Health. (2022) 7:e008793. doi: 10.1136/bmjgh-2022-008793, PMID: 35998978 PMC9402450

[53] Grande M Bjørnsen LP Næss-Pleym LE Laugsand LE Grenne B. Observational study on chest pain during the Covid-19 pandemic: changes and characteristics of visits to a Norwegian emergency department during the lockdown. BMC Emerg Med. (2022) 22:1–11. doi: 10.1186/s12873-022-00612-w 35366802 PMC8976421

[54] Sinkeldam M Buenen AG Celiker E van Diepen M de Vos AM. Characteristics of chest pain in COVID-19 patients in the emergency department. Neth Hear J. (2022) 30:526–32. doi: 10.1007/s12471-022-01730-7, PMID: 36269453 PMC9589604

[55] Islam MA Kundu S Alam SS Hossan T Kamal MA Hassan R. Prevalence and characteristics of fever in adult and paediatric patients with coronavirus disease 2019 (COVID-19): a systematic review and meta-analysis of 17515 patients. PLoS One. (2021) 16:e0249788. doi: 10.1371/journal.pone.0249788, PMID: 33822812 PMC8023501

[56] Zhang PI Hsu CC Kao Y Chen CJ Kuo YW Hsu SL. Real-time AI prediction for major adverse cardiac events in emergency department patients with chest pain. Scand J Trauma Resusc Emerg Med. (2020) 28:1–7. doi: 10.1186/s13049-020-00786-x, PMID: 32917261 PMC7488862

[57] Aerts M Minalu G Bösner S Buntinx F Burnand B Haasenritter J. Pooled individual patient data from five countries were used to derive a clinical prediction rule for coronary artery disease in primary care. J Clin Epidemiol. (2017) 81:120–8. doi: 10.1016/j.jclinepi.2016.09.011, PMID: 27773828

[58] Gencer B Vaucher P Herzig L Verdon F Ruffieux C Bösner S.. Ruling out coronary heart disease in primary care patients with chest pain: a clinical prediction score. BMC Med. (2010) 8:9. doi: 10.1186/1741-7015-8-9 20092615 PMC2832616

[59] Forshey BM Guevara C Laguna-Torres VA Cespedes M Vargas J Gianella A. Arboviral etiologies of acute febrile illnesses in western South America, 2000–2007. PLoS Negl Trop Dis. (2010) 4:e787. doi: 10.1371/journal.pntd.0000787, PMID: 20706628 PMC2919378

[60] Reller ME Wunder EA Jr Miles JJ Flom JE Mayorga O Woods CW. Unsuspected leptospirosis is a cause of acute febrile illness in Nicaragua. PLoS Negl Trop Dis. (2014) 8:e2941. doi: 10.1371/journal.pntd.0002941, PMID: 25058149 PMC4109853

[61] Assiri A Al-Tawfiq JA Al-Rabeeah AA Al-Rabiah FA Al-Hajjar S Al-Barrak A. Epidemiological, demographic, and clinical characteristics of 47 cases of Middle East respiratory syndrome coronavirus disease from Saudi Arabia: a descriptive study. Lancet Infect Dis. (2013) 13:752–61. doi: 10.1016/S1473-3099(13)70204-4, PMID: 23891402 PMC7185445

[62] Cheng VC Chan JF To KK Yuen KY. Clinical management and infection control of SARS: lessons learned. Antivir Res. (2013) 100:407–19. doi: 10.1016/j.antiviral.2013.08.016, PMID: 23994190 PMC7132413

[63] Pollán M Pérez-Gómez B Pastor-Barriuso R Oteo J Hernán MA Pérez-Olmeda M. Prevalence of SARS-CoV-2 in Spain (ENE-COVID): a nationwide, population-based seroepidemiological study. Lancet. (2020) 396:535–44. doi: 10.1016/S0140-6736(20)31483-5 32645347 PMC7336131

[64] Uyoga S Adetifa IM Karanja HK Nyagwange J Tuju J Wanjiku P. Seroprevalence of anti–SARS-CoV-2 IgG antibodies in Kenyan blood donors. Science. (2021) 371:79–82. doi: 10.1126/science.abe1916 33177105 PMC7877494

[65] Kadi N Khelfaoui M. Population density, a factor in the spread of COVID-19 in Algeria: statistic study. Bull Natl Res Cent. (2020) 44:1–7. doi: 10.1186/s42269-020-00393-x, PMID: 32843835 PMC7439635

[66] Khavarian-Garmsir AR Sharifi A Moradpour N. Are high-density districts more vulnerable to the COVID-19 pandemic? Sustain Cities Soc. (2021) 70:102911. doi: 10.1016/j.scs.2021.102911, PMID: 36567891 PMC9760197

[67] Agca H Akalin H Saglik I Hacimustafaoglu M Celebi S Ener B. Changing epidemiology of influenza and other respiratory viruses in the first year of COVID-19 pandemic. J Infect Public Health. (2021) 14:1186–90. doi: 10.1016/j.jiph.2021.08.004, PMID: 34399190

[68] Chow EJ Uyeki TM Chu HY. The effects of the COVID-19 pandemic on community respiratory virus activity. Nat Rev Microbiol. (2023) 21:195–210. doi: 10.1038/s41579-022-00807-9, PMID: 36253478 PMC9574826

[69] Groves HE Papenburg J Mehta K Bettinger JA Sadarangani M Halperin SA. The effect of the COVID-19 pandemic on influenza-related hospitalization, intensive care admission and mortality in children in Canada: a population-based study. Lancet Regional Health Am. (2022) 7:100132. doi: 10.1016/j.lana.2021.100132, PMID: 35291567 PMC8913102

[70] Takeuchi H Kawashima R. Disappearance and re-emergence of influenza during the COVID-19 pandemic: association with infection control measures. Viruses. (2023) 15:223. doi: 10.3390/v15010223, PMID: 36680263 PMC9862942

[71] Lee SS Viboud C Petersen E. Understanding the rebound of influenza in the post COVID-19 pandemic period holds important clues for epidemiology and control. Int J Infect Dis. (2022) 122:1002–4. doi: 10.1016/j.ijid.2022.08.002, PMID: 35932966 PMC9349026

[72] Cheng K Wu C Gu S Lu Y Wu H Li C. WHO declares end of COVID-19 global health emergency: lessons and recommendations from the perspective of ChatGPT/GPT-4. Int J Surg. (2023) 29:10–97. doi: 10.1097/JS9.0000000000000521, PMID: 37246993 PMC10498859

[73] Gupta A Siddiqui F Purwar S Joshi R Mukhopadhyay C. Is it always COVID-19 in acute febrile illness in the tropics during the pandemic? PLoS Negl Trop Dis. (2022) 16:2–4. doi: 10.1371/journal.pntd.0010891, PMID: 36322563 PMC9629600

[74] Phelan AL Sorrell EM Standley CJ Watson C Sauer L Rivers CM. COVID-19 has left the world less prepared for an influenza pandemic. Nat Med. (2023) 29:1044–5. doi: 10.1038/s41591-023-02340-5, PMID: 37076718

