# Correction: Clinical and epidemiological characteristics of influenza and SARS-CoV-2 virus among patients with acute febrile illness in selected sites of Ethiopia 2021–2022

**DOI:** 10.3389/fpubh.2025.1664179

**Published:** 2025-08-04

**Authors:** Musse Tadesse Chekol, David Sugerman, Adamu Tayachew, Zelalem Mekuria, Neamin Tesfay, Aynalem Alemu, Andargachew Gashu, Wolde Shura, Melaku Gonta, Admikew Agune, Aster Hailemariam, Yonas Assefa, Mesfin Wossen, Abdulhafiz Hassen, Parsons Michele, Rachel Silver, Hulemenaw Delelegn, Lozano Briana, Tesfu Kasa, Nigatu Kebede

**Affiliations:** ^1^Public Health Emergency Management Center, Ethiopian Public Health Institute, Addis Ababa, Ethiopia; ^2^Aklilu Lemma Institute of Pathobiology, Addis Ababa University, Addis Ababa, Ethiopia; ^3^U.S. Centers for Disease Control and Prevention (CDC), Atlanta, GA, United States; ^4^Global One Health Initiative (GOHi), Ohio State University, Columbus, OH, United States

**Keywords:** AFI, influenza virus, SARS-CoV-2, Ethiopia, proportion

An incorrect Funding statement was provided. The funding statement in the published article was displayed as “The author(s) declare that no financial support was received for the research and/or publication of this article”. The correct funder is [Centers for Disease Control and Prevention]/the correct funding statement reads:

The author(s) declare that financial support was received for the research and/or publication of this article. The publication was supported by the Cooperative Agreement Number, NU2HGH000069 funded by the Centers for Disease Control and Prevention. Its contents are solely the responsibility of the authors and do not necessarily represent the official views of the Centers for Disease Control and Prevention or the Department of Health and Human Services.

The original version of this article has been updated.

